# Electromechanically reconfigurable plasmonic photodetector with a distinct shift in resonant wavelength

**DOI:** 10.1038/s41378-023-00504-4

**Published:** 2023-03-09

**Authors:** Masaaki Oshita, Shiro Saito, Tetsuo Kan

**Affiliations:** 1 Graduate School of Informatics and Engineering, The University of Electro-Communications, 1-5-1 Chofugaoka, Chofu-city, Tokyo 182-8585 Japan; 2 IMRA JAPAN CO., LTD, 2-36, Hachiken-cho, Kariya, Aichi 448-8650 Japan

**Keywords:** Optical sensors, Electrical and electronic engineering

## Abstract

Plasmonic photodetectors have received increasing attention because their detection properties can be designed by tailoring their metal structures on surfaces without using any additional components. Reconfiguration of the plasmonic resonant state in a photodetector is relevant for various applications, including investigating in situ adaptive detection property changes, depending on the situation, and performing single-pixel spectroscopy in geometrically limited regions. However, the spectral responsivity change with conventional reconfiguration methods is relatively small. Here, we propose a plasmonic photodetector that reconfigures its spectral responsivity with electromechanical deformation instead of bias tuning. The photodetector consists of a gold plasmonic grating formed on an n-type silicon cantilever, and the spectral responsivity is reconfigured by electromechanically scanning at an incident angle to the grating on the cantilever. The photodetector exhibits peak shifts in spectral responsivity in a wavelength range from 1250 to 1310 nm after electromechanical reconfiguration. Finally, for potential future applications, we demonstrate near-infrared spectroscopy using the photodetector. This photodetector has the potential to be adopted as a near-infrared spectrometer in industrial silicon imaging systems because its structure enables subbandgap photodetection on silicon by a Schottky junction.

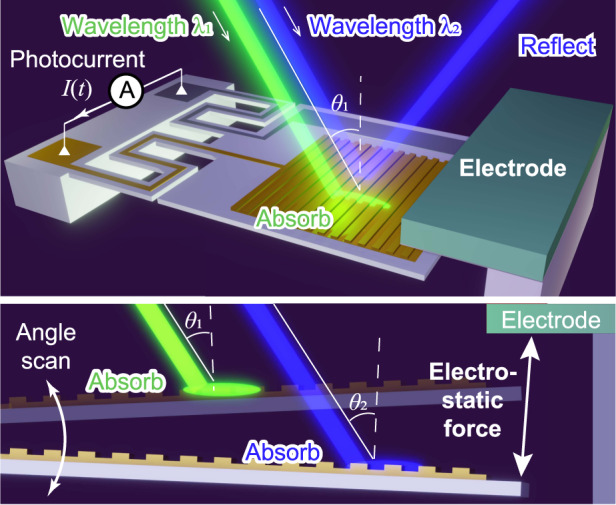

## Introduction

Plasmonic photodetectors have been actively studied because they allow us to design their detection properties, such as spectral or polarization responsivities, by tailoring their metal structures on surfaces without additional optical components^1–10^. The metal structure, called a plasmonic structure, acts as a filter that selectively absorbs the light of a target property. The capability to change the detection properties by reconfiguring the state of the plasmon resonance after photodetector fabrication is applicable not only to in situ adaptive detection property changes, depending on the situation, but also to single-pixel spectroscopy and polarization measurements in geometrically constrained spaces. Recently, several studies have reported dynamically reconfiguring the spectral responsivity of plasmonic photodetectors by applying a bias to materials, including nanocrystals^11^, organic semiconductors^12^, and transition metals^13^. However, the change in the spectral responsivity of these photodetectors is relatively small; in particular, the resonant wavelength has not been shifted continuously at room temperature.

Here, we propose a plasmonic photodetector that reconfigures its spectral responsivity with electromechanical deformation instead of bias tuning. In this study, we observed distinctive peak shifts of the spectral responsivity using our photodetector in the near-infrared range from 1250 to 1310 nm at room temperature. Mechanical reconfiguration for the geometry of plasmonic structures^14–16^ has been extensively proposed because it provides a unique and exciting feature—active control of the color of the reflected light^17^—and varies the spectrum or focal length^18–20^ in comparison to static structures. Despite various emerging studies on mechanical modulation methods, most of these studies have focused on filter^21–27^ and beam steering^17–20,28,29^ applications. Previously, we proposed a plasmonic photodetector with dynamic reconfiguration photodetection properties based on mechanical deformation^30^. In previous work, the peak shifts in the spectral responsivity were observed over a broad wavelength range of 1200–1500 nm when the properties of the photodetector were changed by performing an incident angle scan with a mechanical structure of −21 to 21 deg. However, the air pressure supplied by an external acoustic source was used to actuate the structure, limiting the miniaturization of the entire system. In this study, the plasmonic photodetector is composed of a gold diffraction grating formed on an n-type silicon microelectromechanical system (MEMS) cantilever, and the cantilever is driven by an electrostatic force to electromechanically reconfigure its detection characteristics. The electromechanical drive can be integrated into a MEMS module, shrinking the whole system size. However, the electromechanical drive introduces a displacement current that affects the output signal of the photodetector. The elimination of this displacement current induced by an electromechanical drive is also discussed. Finally, we demonstrate that near-infrared spectroscopy using this reconfiguration method is possible for future photodetector applications.

### Principle of electromechanical reconfiguration for plasmonic photodetection

**Fig. 1:**
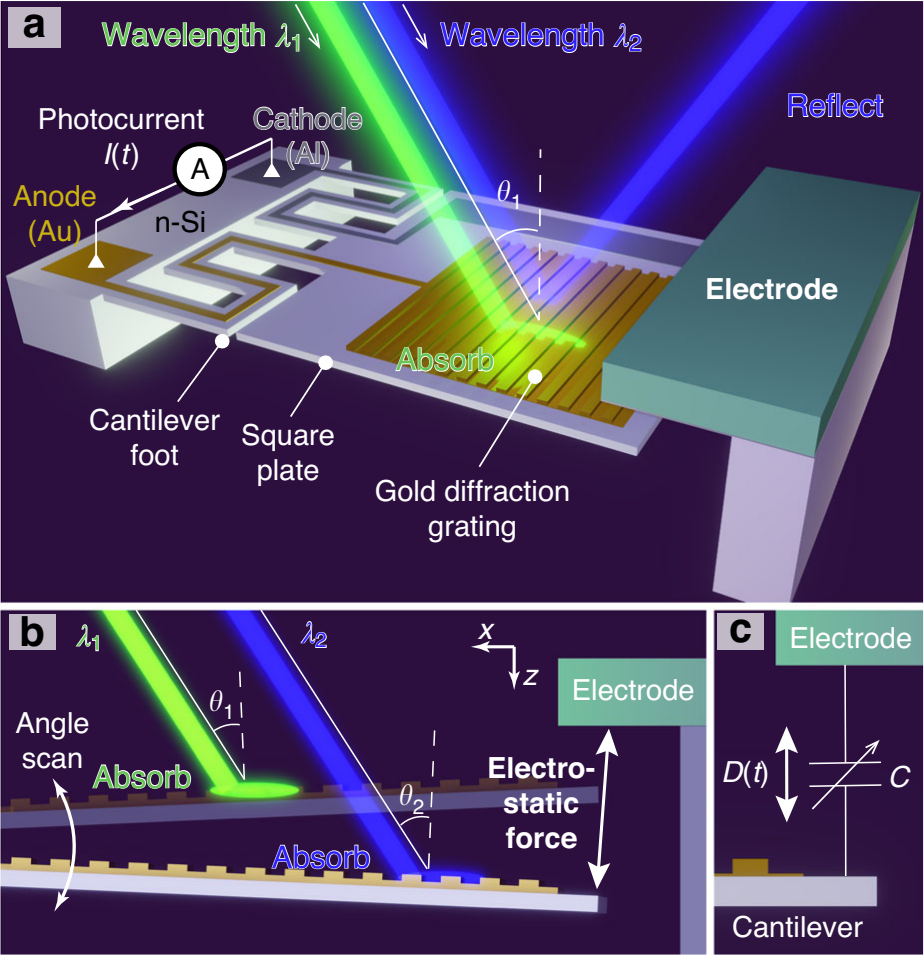
Principle of the electromechanically reconfigurable plasmonic photodetector. **a** Schematic of the reconfigurable photodetector with a plasmonic structure and gold diffraction grating. **b** Angular scanning with electromechanical actuation and wavelength-specific photodetection. **c** Capacitive coupling between the electrode and the cantilever that induces a periodic displacement current *D(t)*

The photodetector used in this study consists of an n-type silicon (n-Si) cantilever with a plasmonic gold diffraction grating formed on its surface and an electrode placed next to it (Fig. 1a).

The electrode is positioned above the cantilever, and an electrostatic force is generated by applying a voltage between the electrode and the cantilever (Fig. 1b). The electrostatic force resonates the cantilever and reconfigures the plasmonic resonant state at an incident angle of light *θ* to the grating. Dual zigzag cantilever feet are used to decrease the stiffness and improve the amplitude of the scanning angle of incidence. When incident light of a specific wavelength enters at a corresponding angle, surface plasmon resonance (SPR) occurs and absorbs the incident light. The light absorbed by SPR supplies energy for gold electrons on the grating, and excited electrons jump over the energy barrier between gold and n-type silicon, called the Schottky barrier. We detected the incident light of a specific wavelength to measure these electrons as a photocurrent *I(t)* through the anode (gold, Au) and the cathode (aluminum, Al). Since the electrostatic force vibrates the incident angle, the detection wavelength of the photodetector is scanned periodically. However, capacitive coupling is inevitably formed between the cantilever and the electrode (Fig. 1c), and a periodic displacement current *D(t)* is superimposed on the photodetector output signal because this capacitive component is repeatedly charged and discharged. We record this periodic displacement current and subtract it from the photodetector output with a differential amplifier. In this study, we first confirm that the electrostatic force from the electrode resonates with the cantilever, and we then measure the optical response of the photodetector via the elimination method for the displacement current. Finally, we perform near-infrared spectroscopy using this reconfiguration method with reconstructive spectroscopy^30,31^.

## Results and discussion

### Angular scanning with an electromechanical drive

The reconfigurable plasmonic photodetector was produced by following the fabrication method described in another reference^30^. We introduce a brief description of the dimensions and fabrication process of the photodetector. The photodetector consists of one 2.5 mm square plate with a gold diffraction grating and an n-type silicon cantilever with zigzag feet. The gold diffraction grating has a depth of approximately 100 nm and a pitch of 3.4 μm. The gold diffraction grating is fabricated by depositing a thin gold layer on the grating structure directly formed on the plate by reactive ion etching (RIE). The cantilever was formed by bulk micromachining using silicon on insulator (SOI) wafers; the thicknesses of the device layer, the buried oxide layer, and the handle layer were 25, 1.5, and 500 μm, respectively. A photograph of the fabricated photodetector is shown in Fig. S1.

**Fig. 2:**
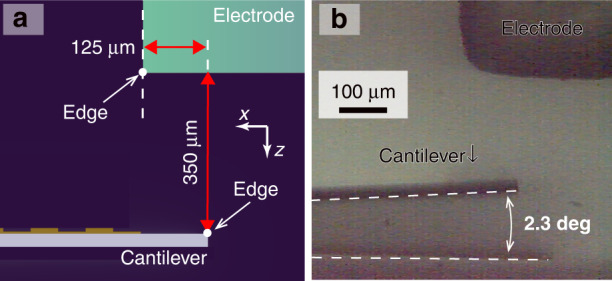
Angular scanning with an electromechanical drive. **a** A schematic of the relative position between the cantilever and electrode from the side view of the setup. **b** A micrograph of the resonating cantilever at the maximum angle of incidence

The angular scanning amplitude obtained via electrostatic drive by the electrode was investigated in the experimental system described in Supplementary Note 1. This setup (Fig. S2a) consists of an electrode on a miniaturized XZ stage and the photodetector. The positional relationship between the cantilever and electrode (Fig. 2a) was obtained by moving the electrode to the cantilever via the XZ stage such that the scanning angle of the cantilever was as large as possible (Supplementary Note 1). We then recorded a high-speed micrograph of the cantilever motion with respect to the electromechanical angular scanning. The micrograph of the cantilever at the maximum displacement is shown in Fig. 2b. From the micrograph, we determined that the amplitude of the scanning angle is approximately ±2.3 deg. If the cantilever is bent at this angle, the maximum deflection in the plate is estimated from the simulation (Fig. S3a) to be 0.33 pm (Supplementary Note 2). This deflection is sufficiently smaller than the grating depth (100 nm), the smallest dimension in a gold grating. Then, we concluded that the change in the spectral response due to the bending of the flat part is negligible.

### Measurements of angular responsivity

**Fig. 3:**
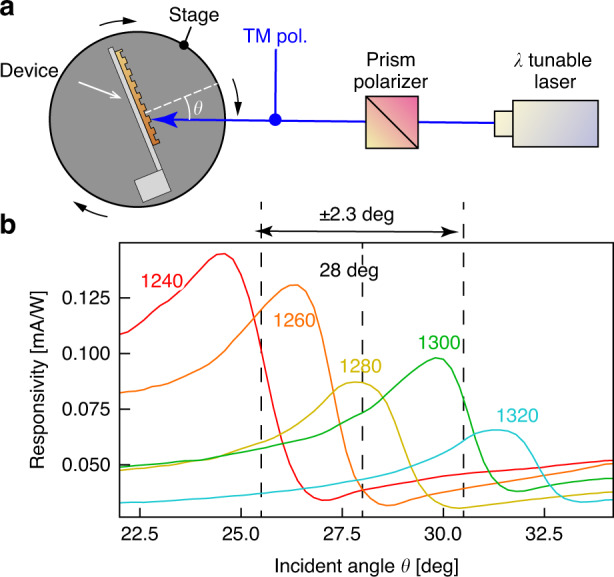
Responsivity at each angle of incidence, obtained by angular scanning with a rotating stage. **a** A schematic of the experimental setup and **b** the responsivities

As mentioned above, the grating of the photodetector exhibits different detection wavelengths at each incident angle. Then, we preliminarily investigated the angular responsivity of the photodetector by scanning an incident angle with a rotation stage without electromechanical actuation. We defined the responsivity *R* described in *R* = *I/P,* where *P* is the intensity of incident light [W], and *I* is the photocurrent [A] that the photodetector outputs. The responsivity of the photodetector has a peak at an angle of incidence where SPR is generated. We, therefore, measured the responsivity in the range of incident angles from 22 to 34 deg, and the resolution of the angle was 0.2 deg. The experimental system for the responsivity measurement is shown in Fig. 3a. The setup was composed of a photodetector, and the electrode was installed on a rotation stage so that the trench of the grating and the rotation axis were parallel (Fig. 3a). Monochromatic near-infrared light from a wavelength-tunable light source (SC-450 and AOTF NIR2, Fianium, U.K.) was converted into transverse magnetic (TM) polarized light via a broadband polarizing beam splitter (PBSW-10-10/20, Sigma Koki, Japan) that illuminated the grating. The full width at half maximum (FWHM) with respect to the wavelength of this monochromatic light was approximately 7 nm. The resulting photocurrent signal was converted into voltage by a transimpedance amplifier (gain 10^7 ^V/A). The converted voltage signal of the photocurrent was measured using a DC voltage current monitor (6242, ADCMT, Japan) for each incident angle *θ* and wavelength *λ*. An incident angle *θ* was scanned by moving the rotational stage. To reduce electrical noise from the surrounding environment, the whole setup was embedded in a shield box while maintaining the positional relationship in Fig. 2a. The shield box possessed an aperture to introduce incident light into the grating of the photodetector. To determine the responsivity of our photodetector, the intensity of the incident light was recorded using a power meter (PM320E-S122C, Thorlabs, U.S.A) for each wavelength beforehand. The responsivities for representative wavelengths are shown in Fig. 3b. Each wavelength is displayed in nm on a corresponding plot. As shown in Fig. 3b, the responsivities had a prominent peak at a unique angle of incidence *θ*, and the diffraction order *m* of these peaks was *m* = −4. These angular positions of peaks systematically changed with the alteration of wavelength, and they were consistent with SPR theory. From these results, we concluded that the fabricated photodetector possessed a unique responsivity at each incident angle, and the peak of the responsivity followed SPR theory.

### Photocurrent measurement during electromechanical reconfiguration

**Fig. 4:**
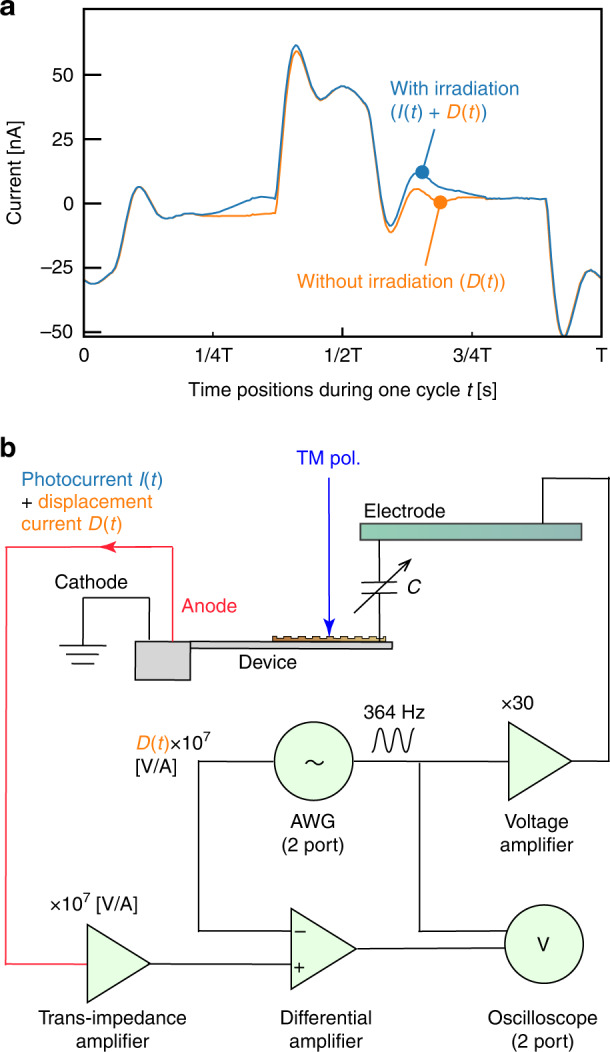
An experimental setup for removing the displacement current. **a** Displacement current signals induced by electromechanical actuation with or without light irradiation, averaged from two cycles of the signal. **b** An experimental setup for removing the displacement current signal by the differential amplifier. The function generator emulates the displacement current signal by generating a prerecorded signal without light irradiation, and the photocurrent is extracted from the mixture signal of the photodetector by the differential amplifier

Next, we measured the photocurrent *I(t)* during electromechanical reconfiguration of the cantilever. First, it should be noted that electromechanical reconfiguration has a drawback: it causes an undesirable displacement current *D(t)* induced by the charge and discharge in capacitive coupling *C* between the cantilever and the electrode (Fig. 1c). To compare the photocurrent and the displacement current, we measured the photocurrent signal with an oscilloscope (TBS 1052B-EDU, Tektronix, U.S.A.) after being amplified by a transimpedance amplifier (gain: 10^7 ^V/A). The anode of the photodetector was connected to the oscilloscope through the amplifier, and the cathode was connected to the ground voltage. We then applied a sinusoidal voltage and recorded the output signal while the cantilever was resonating. The recorded signal (Fig. 4a orange line) exhibited periodicity (T = 2.7 ms = 1/364 Hz) at a frequency of the applied voltage, and the amplitude of this signal was approximately 50 nA. We then irradiated the grating with monochromatic near-infrared light (wavelength: 1300 nm, light intensity: 0.18 mW) and recorded the signal from the photodetector. As shown in Fig. 4a, the mixture of *I(t)* and *D(t)* (Fig. 4a blue line) differs from the displacement current by only approximately 15 nA. To extract the photocurrent *I(t)* from this mixture signal, a numerical extraction method that subtracts the recorded displacement current *D(t)* from the mixture signal numerically is an appropriate candidate. However, this numerical extraction method suffers from a more significant effect of quantization error since the vertical resolution of the signal is inversely proportional to the dynamic range. The oscilloscope should be used only to measure the photocurrent signal extracted from the mixture signal with a port of the oscilloscope. Previous experiments and results (Fig. 4a) have shown that the displacement current is periodic and synchronized with the applied voltage signal from the electrode. Therefore, we initially record the displacement current without light irradiation and then extract only the photocurrent from the mixture signal with light irradiation by subtracting the recorded displacement current formed from an arbitrary waveform generator (AWG).

Then, we constructed the experimental system shown in Fig. 4b to extract the photocurrent from the mixture signal. The mixture signal through a transimpedance amplifier entered a noninverting port, denoted as +, of the differential amplifier (5307, NF Corporation, Japan). The previously recorded signal without light irradiation was provided by an AWG (33500B, Keysight, U.S.A.), and this signal entered an inverting port, denoted as −, of the differential amplifier. If the signal from the AWG did not match the phase of the displacement current, the signal phase from the AWG was adjusted manually during differential amplification without light irradiation so that the amplitude of the differentiated signal was as small as possible. The extracted photocurrent signal was measured with an oscilloscope.

**Fig. 5:**
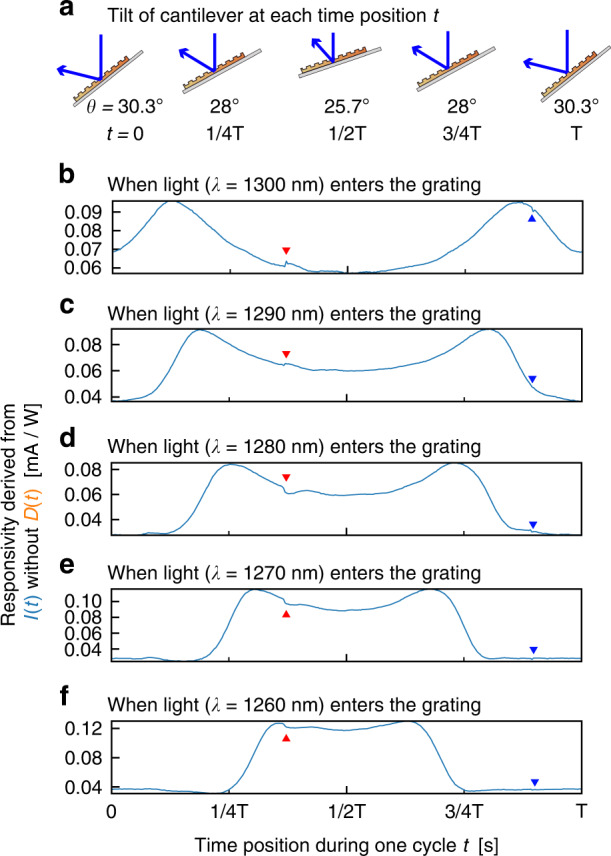
Responsivities of our photodetector on electromechanical scanning. **a** The scanning angle of the cantilever at each time position. **b**–**f** The responsivities for each time position during one cycle of the obtained signals, averaged from two cycles of the signal

For a comparison of the responsivity obtained by the rotation stage (Fig. 3b) and the electromechanical drive, the initial value of the incident angle was adjusted to 28 deg at the center of the angular range, as shown in Fig. 3b. However, since the amplitude of the scanning angle with the electromechanical drive is only ±2.3 deg, the electromechanical drive scans only in the region indicated by a double arrow spanning from 25.7 deg to 30.3 deg in Fig. 3b. We calculated the responsivities of the photodetector, which excluded the contribution of the displacement current. The responsivity of the photodetector is shown in Fig. 5b–f. Figure 5a shows the scanning angle of the cantilever in this experiment at time *t* of 0, T/4, T/2, 3 T/4, and T. The displayed signals in Fig. 5b–f clearly show the elimination of the displacement current. In the timing range from 1/2 T to T corresponding to the scanning angle from 25.7 deg to 30.3 deg, the incident angle at the peaks of the responsivity shown in Fig. 5b–f increases with the wavelength of the incident light, and these peak shifts are coherent with Fig. 3b. Then, we concluded that the photocurrent measurement of the photodetector could be performed during electromechanical reconfiguration using our elimination method for the displacement current.

A slight notch-like artifact in Fig. 5b–f is shown at 3 T/8 (indicated by a red triangle) and 7/8 T (indicated by a blue triangle). Small lags or errors between the displacement current signal and the signal from the AWG may cause these artifacts. The lag or error could be decreased by improving the time and voltage resolution of the AWG or with careful manual adjustment. In the future, the displacement current from a nonphotosensitive cantilever near the photodetector will be exploited to eliminate these artifacts as an inverting signal of differential amplification.

### Spectroscopic demonstration

**Fig. 6:**
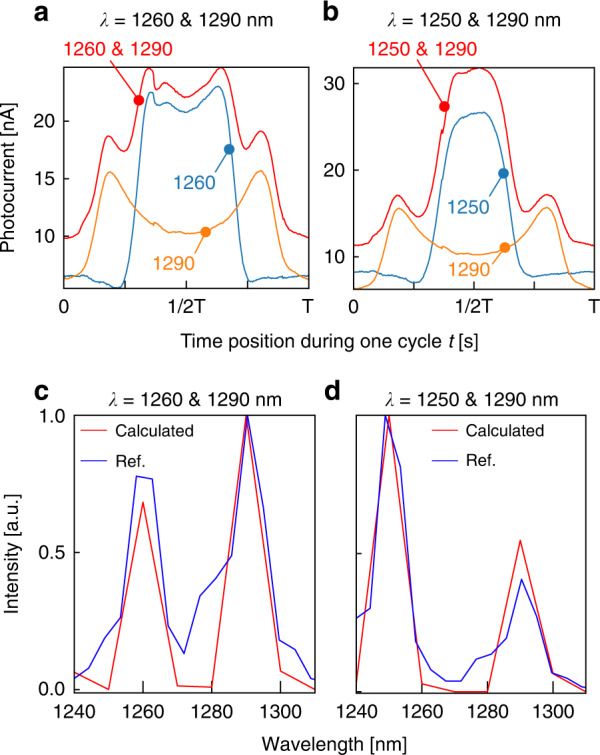
Demonstration of spectroscopy. **a**, **b** Photocurrent signals measured for spectroscopy, generated with irradiation of two monochromatic wavelengths depicted by the red line. The orange and blue lines indicate the photocurrent signals with monochromatic irradiation. Each wavelength is displayed in nm on a corresponding plot. **c**, **d** Spectra derived from the photocurrent signals acquired with our photodetector and the removal method for the displacement current. The red line illustrates the spectra of our method; the blue line displays the results obtained from the reference near-infrared spectrometer (Sol2.2 A, BWTEK, U.S.A.)

Finally, we performed spectroscopic measurements using the electromechanical tunability of our photodetector to demonstrate its spectroscopic application. The detailed spectroscopic measurement method in which the plasmonic photodetector is used without electromechanical reconfiguration is explained in other references^30,31^. Two monochromatic wavelengths ((a) λ = 1260 and 1290 nm, (b) λ = 1250 and 1290 nm) simultaneously entered the grating from the light source, and the photocurrent signals obtained during electromechanical reconfiguration are depicted by the red line in Fig. 6a, b. When performing spectroscopy, the photocurrent obtained by using simultaneous input is assumed to be a linear sum of the photocurrents generated by each wavelength. Therefore, we show the signal with monochromatic irradiation in Fig. 6a, b for comparison with the signal generated by simultaneous irradiation. Figure 6a, b shows that the peaks of the signals with the irradiation of two monochromatic lights are consistent with the peak originating from each monochromatic irradiation. Therefore, we confirmed that the photocurrent obtained by using simultaneous input is a linear sum of the photocurrent generated by each wavelength, even under an electrostatic drive.

Next, using these photocurrent signals in Fig. 6a, b and the spectroscopic calculation method in other references^30,31^, we calculated the spectra shown in Fig. 6c, d. The red lines in Fig. 6c, d show the results calculated from the signals from our photodetector, and the blue lines show the results of a reference spectrometer (Sol2.2 A, BWTEK, U.S.A.). As illustrated in Fig. 6c, the spectrum calculated from the signal of our photodetector has peaks at 1260 and 1290 nm, and it was clear that the spectrum obtained from the reference spectrometer had peaks at the same wavelength positions. However, the spectrum calculated from the photodetector signal also shows a peak at 1240 nm. This peak might be derived from the notch-shaped artifact in Fig. 5b–f. We expect that this peak could be removed by improving the removal method for the displacement current signal. The same assessment holds for the results shown in Fig. 6d. Since the number and wavelength of peaks in Fig. 6d are also identical, we thus confirmed that the spectra derived from our photodetector are consistent with the results of a reference spectrometer, even if the spectra have different intensities at each wavelength. As shown in Fig. 6c, d, the peak wavelengths of the spectra are only 1250 nm and 1260 nm apart, indicating that this spectroscopy can discriminate a 10 nm wavelength difference. Figure 6c also shows that our method can discriminate a wavelength gap of at least 30 nm. Moreover, we conclude that an electromechanically reconfigurable plasmonic photodetector could perform spectroscopy in the wavelength range from 1240 nm to 1310 nm with 30 nm wavelength resolution by using the elimination method for the displacement current. A reconfigurable plasmonic photodetector previously proposed by authors, which changes its detection characteristics with acoustic pressure, has been demonstrated to be capable of spectroscopy in the wavelength range from 1230 nm to 1330 nm with a wavelength resolution of 20 nm^32^; therefore, our photodetector may be able to achieve these performances by revising the plasmonic structure and the electrostatic mechanism in principle.

## Conclusion

In conclusion, we propose an electromechanically reconfigurable plasmonic photodetector driven by an electrostatic actuator to provide a significant resonant wavelength shift. The photodetector structure is composed of an n-type silicon cantilever combined with a plasmonic gold grating and an electrode placed above the cantilever. Since the gold diffraction grating absorbs a specific wavelength of light at the theoretically corresponding angle of incidence, the responsivity of the plasmonic photodetector has peaks at the angle of incidence. The cantilever resonates when an electrostatic force is generated by applying a voltage between the cantilever and the electrode. As the cantilever resonates, the angle of incidence to the grating changes periodically, reconfiguring the detection wavelength. In this study, the electrodes are placed outside the photodetector to verify whether the output current can be obtained while the photodetector is driven electrostatically. To maximize the scanning angle by electromechanical actuation, we aligned the edge of the cantilever at a relative distance from the surface of the electrode to 125 μm in front and 350 μm in height (Fig. 2a). A sinusoidal voltage of 364 Hz and 150 ± 150 V was applied between the electrode and the cantilever, and we obtained a ±2.3 deg scanning angle of the cantilever (Fig. 2b). The photocurrent signal from the photodetector during electromechanical actuation was recorded by subtracting the displacement current signal in the experimental setup of Fig. 4b at room temperature. Because the peak shifts in Fig. 5b–f were consistent with the results of the scanning angle on the rotation stage (Fig. 3b) from 25.4 deg to 30.3 deg, we assume that these peaks were due to SPR generation. From these results, we conclude that the spectral responsivity of our photodetector exhibits distinctive peak shifts in the near-infrared range from 1250 to 1310 nm. For a demonstration of potential future applications, we performed near-infrared spectroscopy using our photodetector with the photocurrent extraction method. As shown in Fig. 6c, d, these spectra were comparable to those from the reference spectrometer. From these results, we conclude that a reconfigurable plasmonic photodetector can perform spectroscopy in the wavelength range from 1240 nm to 1310 nm with electromechanical scanning of the cantilever by using the elimination method for the displacement current signal.

We believe that the photodetector has the potential to extend its wavelength range to longer wavelengths in the mid-infrared. One way to extend the wavelength range is to change the type of metal used in the Schottky junction of the photodetector. Our photodetector detects near-infrared light in a range of 1240 to 1310 nm as a photocurrent using a Schottky barrier formed between gold and n-type silicon. We can use another metal with a lower work function than gold to achieve a lower Schottky barrier height for extending the cutoff wavelength. Our team has successfully extended the cutoff wavelength to 3.4 μm by using a Si/PtSi junction^33^. We estimate that the extension of the wavelength range is limited to approximately 10 μm at room temperature because the lower limit of the barrier height is 0.026 eV due to thermal energy. Furthermore, our mechanical reconfiguration method of photodetectors can be applied to industrial silicon devices by addressing several technical issues, such as shrinking the footprint of the photodetector and enlarging the amplitude of angular scanning. Angular scanning with this structure has a smaller scanning angle range than other MEMS angle scanners^34,35^. Increasing the amplitude of the angular scanning leads to an expansion of the spectral range for spectroscopic applications and an improvement in the field of view (FOV) for the application of single-pixel imaging. We expect that the amplitude of the angular scanning can be improved by integration into the digital micromirror device (DMD), which is currently available with an angular scanning structure of ±12 deg by applying 17 V as the driving voltage and a pixel size of 8 μm square^35^. This research also supports the possibility of integrating plasmonic photodetectors and other electrostatically driven micromechanical structures. This technology expands the range of future plasmonic photodetector applications.

## Supplementary information

Supplementary Information
