# Supplementary material for: Electromechanically reconfigurable plasmonic photodetector with a distinct shift in resonant wavelength
Source: Microsyst Nanoeng. 2023 Mar 9;9:26. doi: 10.1038/s41378-023-00504-4 (PMC9998386; doi:10.1038/s41378-023-00504-4)
Supplement: Supplementary file 1 — Supplementary Information [file 41378_2023_504_MOESM1_ESM.docx]

Electromechanically reconfigurable plasmonic photodetector with a distinct shift of resonant wavelength: supplemental document

Supplementary Note 1.

Experimental setup for electromechanical deformation of the photodetector and approaching the electrode to the cantilever

For the electrostatic drive of our plasmonic photodetector (Figure S1), we constructed the experimental system shown in Figure S2(a). The electrode was fabricated using a 3D printer, and aluminum was deposited on the backside of the printer at approximately 100 nm for continuity. The electrode was fixed on a manual XZ stage (MDE268, Elliot Scientific, Australia) such that the positional relationship between the photodetector and the electrode could be finely adjusted (Figure S2(a)). The amplitude of the angular scanning critically depends on the gap between the photodetector and the electrode along not only the z-axis but also the x-axis, so we employed the XZ stage for fine positioning along both axes. The XZ stage with the electrode and the photodetector is installed on a five mm-thick steel plate to maintain the relative positioning of these two parts. To apply a voltage between the electrode and the cantilever, we wired the electrode for a high-voltage source (M-2620A, Mess-tek, Japan) and the cathode of the photodetector for the ground voltage. The anode of the photodetector had no connections to any circuits during electromechanical actuation. A photograph of the setup composed of the photodetector and the electrode is shown in Figure S2(b). As illustrated in Figure S2b, the electrode (Figure S2(c)) was fixed on the XZ stage (Figure S2(d)).

Next, the relative distance between the edge of the cantilever and the edge of the electrode was measured using a high-speed microscope (VW-5000, Keyence, Japan) from the side of the setup (Figure S2(b)). A schematic of the positional relationship between the cantilever and the electrode is depicted in Figure 2(a). The electrode is shown at the top of the schematic, and the cantilever is at the bottom. The XZ stage adjusted the position of the edge of the electrode along the x-axis to +125 μm from the edge of the cantilever (Figure 2(a)). This amount of movement was limited because the electrode blocks the incident light if the electrode covers the surface of the photodetector. The cantilever presented angular scanning motion when the sinewave voltage (Amplitude: 150±150 [V]) was applied between the electrode and the cantilever. The amplitude of the scanning angle was maximized by adjusting the applied frequency to 364 Hz. We then brought the electrode closer to the cantilever along the z-axis to enlarge the electrostatic force between the electrode and cantilever to increase the amplitude of the scanning angle without contact between the electrode and the cantilever. However, if the electrode and the cantilever are too close, the cantilever may be pulled in on the electrode. The approach along the z-axis ended when the height from the horizontal surface of the electrode to the edge of the cantilever reached 350 μm (Figure 2(a)).


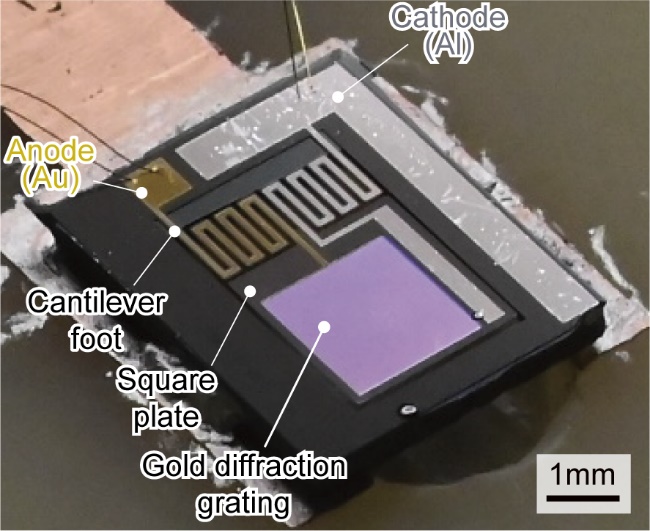


Figure S1. Photograph of the fabricated photodetector used in this paper.


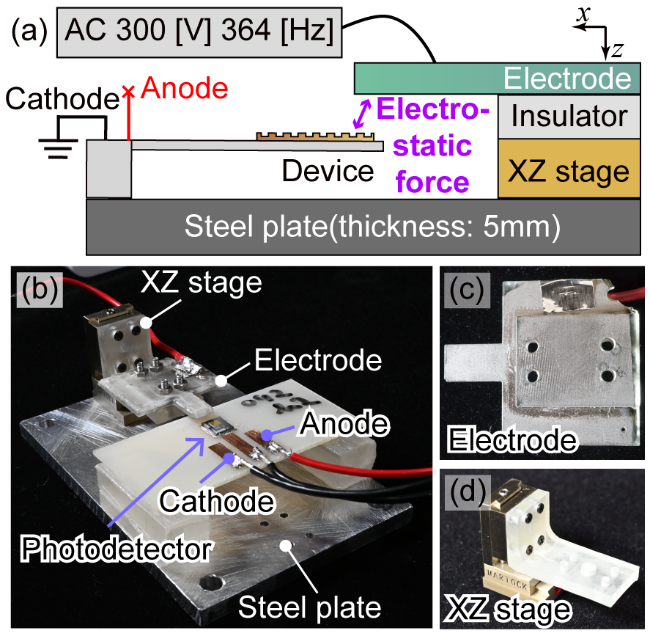


Figure S2 An experimental setup for electroactuation for the reconfigurable plasmonic photodetector. (a) Schematic, (b) photograph of the setup, (c) photograph of the XZ stage, and (d) the fabricated electrode.

Supplementary Note 2.

Simulation of the photodetection area bending

We conducted a finite element method simulation (COMSOL Multiphysics) to investigate whether small bending changes the spectral response of our photodetector. Figure S3(a) shows a cross-sectional surface profile of the cantilever from the root to the tip of the cantilever at the resonant vibration. The sectional line position on the device is also shown in the upper left of Figure S3(b). The area from 0 μm to 1280 μm corresponds to the zigzag foot of the cantilever. The space of the lines around 1280 μm corresponds to the cantilever’s gap between the zigzag foot and the cantilever body. Then, the rest of the profile corresponds to the plate of the photodetector. Figure S3(a) shows that almost all bending took place at the zigzag feet region, and the plate section, which is responsible for photodetection, is almost linear and hardly bent. Next, we calculated the curvature of the plate section to estimate whether the cantilever plate did not bend quantitatively. The result is shown in Figure S3(c). As shown in Figure S3(c), the curvature has a maximum value of 0.23 m^-1^ at 2000 µm from the root, excluding the edge of the plate, and the radius of curvature is 4.4 m. Therefore, we estimated that the amount of deformation in the grating pitch (3.4 µm) is the camber of the arc with the calculated radius (4.4 m). If the grating pitch is regarded as the arc’s chord, the deformation amount is estimated at 0.33 pm. The amount of deformation is tiny compared to the pitch of the grating (3.4 µm) and the height of the groove (100 nm), which is related to the SPR coupling. Therefore, we concluded that the change in the spectral response due to the bending of the flat part is negligible.


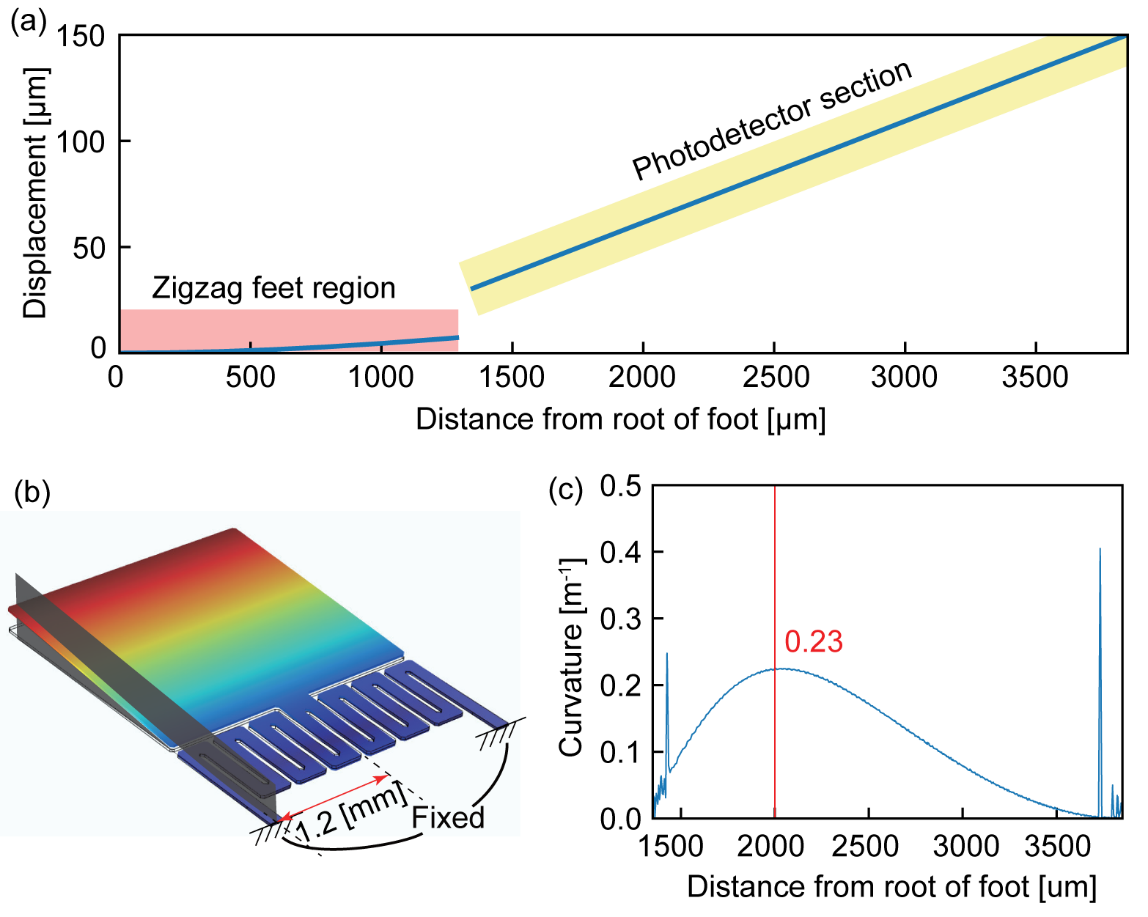


Figure S3 Displacement of the cantilever of the proposed device. (a) A plot of displacement along the distance from the root of the foot. (b) The sectional line position on the device. (c) The curvature of displacement.
